# Efficacy of CagriSema for Reaching Anthropometric Treatment Targets and Cardiometabolic Outcomes: A Secondary, *Post hoc* Analysis of REDEFINE 1

**DOI:** 10.1111/dom.71134

**Published:** 2026-07-26

**Authors:** Luca Busetto, Cynthia Osorto Contreras, Mikkel Hovden Christensen, W. Timothy Garvey, Deborah B. Horn, Barbara McGowan, Claire Prener Miller, Volker Schnecke, Carel W. le Roux

**Affiliations:** ^1^ Department of Medicine University of Padova Padova Italy; ^2^ Novo Nordisk A/S Søborg Denmark; ^3^ University of Alabama at Birmingham Birmingham Alabama USA; ^4^ Center for Obesity Medicine and Metabolic Performance University of Texas McGovern Medical School Houston Texas USA; ^5^ Guy's and St. Thomas' NHS Foundation Trust London UK; ^6^ Diabetes Complications Research Centre Conway Institute, University College Dublin Dublin Ireland; ^7^ Diabetes Research Centre Ulster University Coleraine UK

**Keywords:** clinical trial, obesity care, obesity therapy, weight management

## Abstract

**Aims:**

To assess the added value of absolute anthropometric targets alongside percentage weight loss in the clinical management of obesity.

**Materials and Methods:**

The phase 3a, 68‐week REDEFINE 1 trial randomised adults without diabetes with BMI ≥ 30 kg/m^2^, or ≥ 27 kg/m^2^ with ≥ 1 obesity‐related complication, to once‐weekly CagriSema 2.4 mg/2.4 mg, semaglutide 2.4 mg, cagrilintide 2.4 mg, or placebo, plus lifestyle intervention. This secondary, *post hoc* analysis assessed the proportions of participants achieving BMI < 27 kg/m^2^ and/or WHtR < 0.53 targets, and percentage weight loss by anthropometric target. The association between the proportion of participants maintaining or achieving normalisation of four cardiometabolic outcomes: normoglycemia (glycated haemoglobin < 5.7% and fasting plasma glucose < 5.6 mmol/L); blood pressure (< 130/80 mmHg); triglycerides (< 1.7 mmol/L); lipids (high‐density lipoprotein cholesterol ≥ 1.3 mmol/L [female] or ≥ 1.0 mmol/L [male]) and reaching anthropometric targets or change in body weight (%) was also assessed.

**Results:**

The proportion of participants achieving both BMI < 27 kg/m^2^ and WHtR < 0.53 targets at week 68 was 30.3%, 19.1%, 9.0%, and 3.3% in participants who received CagriSema, semaglutide, cagrilintide, or placebo respectively. Both BMI and WHtR performed similarly as indicators for all four cardiometabolic outcomes. At more stringent cut‐off values, anthropometric targets were better measures than percentage weight loss.

**Conclusions:**

The proportion of participants achieving anthropometric targets was greater with CagriSema Versus other treatments. These findings support further validation of BMI and WHtR anthropometric treatment targets and indicate a potential for indicating amelioration of clinical outcomes in obesity and a greater emphasis on target‐based treatment strategies.

## 1 Introduction

Obesity is a chronic metabolic disease that substantially increases the risk of developing obesity‐related complications and diseases (ORCDs) [1, 2]. In clinical practice, monitoring the efficacy of treatments for chronic diseases often focuses on reaching treatment targets associated with a reduced risk of morbidity or mortality [1], rather than relying on percentage improvements in a specific endpoint. Blood pressure and low‐density lipoprotein (LDL) cholesterol targets are used in clinical practice to ameliorate cardiovascular (CV) risk [1, 3], while reaching glycated haemoglobin (HbA_1c_) targets reduces the risk of developing microvascular complications in type 2 diabetes (T2D) [4]. Advances in the understanding of obesity as a chronic disease have thus led to a shift in obesity management, beyond weight loss, to provide a range of benefits including the prevention and treatment of obesity complications and reduced mortality [1, 5, 6]. However, a lack of widely accepted treatment targets to guide decision‐making remains [6].

Evaluating the efficacy of obesity medications in randomised control trial settings consistently focuses on percentage body weight loss as the primary endpoint [7, 8, 9]. Obesity guidelines typically recommended a treatment target of body weight reductions from ≥ 5% to ≥ 20% depending on patient starting weight and therapy goals [10, 11, 12, 13]. The achievement of a > 5% body weight reduction remains a regulatory requirement to assess the efficacy of an obesity medication [9, 11]. Clinical guidelines, including Canadian [14] and the recently released American Diabetes Association standards of care [13], recommend an individualised approach, with a focus on health outcomes without provision of absolute targets. The European Association for the Study of Obesity framework and American Association of Clinical Endocrinology both recommend an individualised approach for obesity management without provision of absolute targets [15, 16]. Many people with obesity who currently benefit from highly effective treatments, such as the glucagon‐like peptide‐1 (GLP‐1) analogue, semaglutide, or the dual glucose‐dependent insulinotropic polypeptide (GIP) and GLP‐1 receptor agonist tirzepatide, achieve clinically relevant reductions in body weight greatly exceeding the 5% threshold [17, 18]. However, despite such highly efficacious pharmacotherapies, many patients have excess adiposity post‐treatment. Moreover, for any given percentage weight loss there is heterogeneity regarding resulting phenotypic variation, treatment response or body composition and fat distribution. Although there are currently no universally accepted anthropometric targets for obesity, a body mass index (BMI) ≤ 27 kg/m^2^ and waist‐to‐height ratio (WHtR) ≤ 0.53 have been proposed as they are associated with markedly lower risk of ORCDs, encompassing a range of pathophysiologies, including T2D, hypertension, osteoarthritis, and atherosclerotic cardiovascular disease [19], thus creating a state of low disease activity.

In the phase 3a REDEFINE 1 trial (NCT05567796), once‐weekly subcutaneous (s.c) administration of a fixed‐dose combination of cagrilintide 2.4 mg and semaglutide 2.4 mg (CagriSema 2.4 mg/2.4 mg) led to a statistically significant and superior weight loss Versus monotherapies and placebo in participants with a BMI ≥ 27 kg/m^2^, with an estimated mean percent change in body weight from baseline to week 68 of −22.7% Versus −2.3% for placebo, assuming no treatment discontinuation or use of rescue medication [20]. This secondary, *post hoc* analysis of REDEFINE 1 examined the proportion of participants who achieved BMI < 27 kg/m^2^ and WHtR < 0.53 treatment targets, and assessed the percentage of patients achieving a state of low disease activity in participants who achieved or did not achieve BMI and WHtR targets.

## 2 Materials and Methods

### 2.1 Study Design and Participants

REDEFINE 1 (NCT05567796) was a phase 3a, randomised, double‐blind, placebo—and active‐controlled, multi‐centre trial conducted in 22 countries from November 1, 2022, to June 28, 2023, with a 2 year off‐treatment extension period due to end October 19, 2026; full methodology has been published previously [20]. Briefly, adults with a BMI ≥ 30 kg/m^2^, or ≥ 27 kg/m^2^ with ≥ 1 obesity‐related complication were included. Participants were randomised 21:3:3:7 to receive once‐weekly s.c. CagriSema 2.4 mg/2.4 mg, semaglutide 2.4 mg, cagrilintide 2.4 mg, or placebo, and lifestyle intervention in all study groups, for 68 weeks (Figure S1). Key exclusion criteria were diabetes, HbA_1c_ ≥ 6.5% (48 mmol/mol) or a self‐reported change in body weight > 5% within 90 days before screening. Sex, race and ethnicity were self‐reported by participants. REDEFINE 1 was conducted in accordance with the Declaration of Helsinki and the International Conference on Harmonisation of Technical Requirements for Pharmaceuticals for Human Use Good Clinical Practice guidelines. Institutional review board ethics approval was obtained at each study site, and all participants provided written, informed consent to participate in the trial [20].

### 2.2 Endpoints

This secondary, *post hoc* analysis of REDEFINE 1 assessed the proportions of participants achieving BMI < 27 kg/m^2^ and/or WHtR < 0.53 targets in each treatment group, and the percentage weight loss and change in waist circumference (cm) by anthropometric target. The proportion of participants achieving BMI < 27 kg/m^2^ and/or WHtR < 0.53 targets was also assessed by baseline BMI group (≤ 35 kg/m^2^, > 35 kg/m^2^) in each treatment group. In addition, the proportion of participants maintaining or achieving each of the following four cardiometabolic outcomes was assessed in those with respective measurements at baseline and week 68: normoglycaemia, defined as HbA_1c_ < 5.7% and fasting plasma glucose (FPG) < 5.6 mmol/L (100 mg/dL); systolic blood pressure (SBP) < 130 mmHg and diastolic blood pressure (DBP) < 80 mmHg; triglycerides < 1.7 mmol/L (< 150 mg/dL); high‐density lipoprotein (HDL) cholesterol ≥ 1.0 mmol/L (male) or ≥ 1.3 mmol/L (female). The performance of BMI < 27 kg/m^2^ and WHtR < 0.53 anthropometric thresholds and change in body weight (%) in reaching all four cardiometabolic outcomes at week 68 was assessed. The proportion of participants reaching a high‐sensitivity C‐reactive protein (hsCRP) of < 2 mg/L at week 68 was also assessed, alongside the proportion of participants who met all four cardiometabolic outcomes plus hsCRP < 2 mg/L.

### 2.3 Statistical Analyses

The trial‐product estimand was used to assess the effects if treatment was taken as intended with no initiation of rescue intervention (other weight management drugs, devices, or weight loss surgery), and was based on a marginal logistic regression model, using data from the on‐treatment period. Missing and off‐treatment data were imputed assuming missing at random. For the trial product estimand, continuous endpoints were analysed using a mixed model for repeated measurements with restricted maximum likelihood estimation, assuming a missing at random (MAR) mechanism for missing data. The model incorporated treatment as a factor and baseline body weight as a covariate, both nested within visit. Binary endpoints were analysed using marginal logistic regression, with treatment as a factor and baseline body weight as a covariate. Missing data for the marginal logistic regression were addressed through a multiple imputation approach applied separately within each treatment arm, also assuming a MAR mechanism within each treatment arm. Estimated treatment differences (ETDs) are presented with two‐sided 95% confidence intervals (CIs). Statistical analyses were conducted using R statistical software (version 4.3.1).

## 3 Results

### 3.1 Participant Demographics and Baseline Characteristics

In the REDEFINE 1 trial, 3417 participants were randomised to CagriSema 2.4 mg/2.4 mg (*n* = 2108), semaglutide 2.4 mg (*n* = 302), cagrilintide 2.4 mg (*n* = 302), or placebo (*n* = 705), and lifestyle intervention, for 68 weeks (Figure S1) [20]. At baseline, mean (standard deviation [SD]) age was 47.0 (11.8) years, 67.6% of participants were female, and 72.0% were White. Mean (SD) BMI was 37.9 (6.7) kg/m^2^, HbA_1c_ was 5.5 (0.4) % and total and HDL cholesterol were 5.0 (1.0) mmol/L and 1.3 (0.3) mmol/L respectively. Mean baseline SBP was 127.1 (14.2) mmHg, and DBP was 82.1 (9.2) mmHg.

### 3.2 Anthropometric Treatment Targets

Demographics and baseline characteristics by anthropometric target group (BMI < 27 kg/m^2^ and WHtR < 0.53, and BMI ≥ 27 kg/m^2^ and WHtR ≥ 0.53) are shown in Table S1. For observed data from the on‐treatment period, the proportion of participants at week 68 achieving a BMI < 27 kg/m^2^ was 41.0% (592/1444), 24.5% (54/220) and 13.5% (30/223) in each treatment group of participants who received CagriSema 2.4 mg/2.4 mg, semaglutide 2.4 mg or cagrilintide 2.4 mg, respectively, Versus 6.0% (27/451) for placebo (Table 1). For participants achieving a WHtR < 0.53, the proportions for CagriSema 2.4 mg/2.4 mg, semaglutide 2.4 mg, cagrilintide 2.4 mg and placebo groups were 34.8% (503/1444), 25.0% (55/220), 10.8% (24/223) and 6.4% (29/451) respectively. The proportion of participants achieving both BMI < 27 kg/m^2^ and WHtR < 0.53 targets at week 68 was 30.3% (437/1444), 19.1% (42/220) and 9.0% (20/223) in participants who received CagriSema 2.4 mg/2.4 mg, semaglutide 2.4 mg, or cagrilintide 2.4 mg respectively, Versus 3.3% (15/451) for placebo (Table 1).

**TABLE 1:** The proportion of participants achieving anthropometric targets and cardiometabolic outcomes at baseline and week 68.

	CagriSema 2.4 mg/2.4 mg	Semaglutide 2.4 mg	Cagrilintide 2.4 mg	Placebo
	*n* (%)	*n* (%)	*n* (%)	*n* (%)
Number of participants with data at baseline	1860	276	265	535
Number of participants with data at week 68	1444	220	223	451
Anthropometric targets				
BMI < 27 kg/m^2^				
Baseline	0	0	0	3 (0.6)
At week 68	592 (41.0)	54 (24.5)	30 (13.5)	27 (6.0)
WHtR < 0.53				
Baseline	5 (0.3)	2 (0.7)	1 (0.4)	5 (0.9)
At week 68	503 (34.8)	55 (25.0)	24 (10.8)	29 (6.4)
BMI < 27 kg/m^2^ and WHtR < 0.53				
Baseline	0	0	0	0
At week 68	437 (30.3)	42 (19.1)	20 (9.0)	15 (3.3)
Cardiometabolic outcomes				
Normoglycaemia (HbA_1c_ < 5.7% and FPG < 5.6 mmol/L)				
Baseline	843 (45.3)	132 (47.8)	109 (41.1)	233 (43.6)
At week 68	1298 (92.1)	194 (89.4)	129 (59.2)	225 (50.8)
SBP/DBP < 130/80 mmHg				
Baseline	637 (34.2)	97 (35.1)	91 (34.3)	163 (30.5)
At week 68	882 (61.1)	135 (61.4)	102 (45.9)	160 (35.5)
Triglycerides < 150 mg/dL				
Baseline	1312 (70.5)	197 (71.4)	191 (72.1)	365 (68.2)
At week 68	1308 (92.0)	186 (85.7)	180 (81.1)	319 (71.2)
HDL cholesterol ≥ 1.0 mmol/L (M), or ≥ 1.3 mmol/L (F)				
Baseline	1103 (59.3)	176 (63.8)	148 (55.8)	342 (63.9)
At week 68	1092 (76.6)	162 (74.7)	160 (72.1)	293 (65.4)
All four cardiometabolic outcomes				
Baseline	178 (9.6)	27 (9.8)	24 (9.1)	43 (8.0)
At week 68	593 (41.1)	85 (38.5)	37 (16.6)	57 (12.6)

*Note:* Observed data from the on‐treatment period regardless of discontinuation or receipt of rescue medication, for treatment completers.

Abbreviations: BMI: body mass index; DBP: diastolic blood pressure; F: female; FPG: fasting plasma glucose; HbA_1c_: glycated haemoglobin; HDL: high‐density lipoprotein; M: male; SBP: systolic blood pressure; WHtR: waist‐to‐height ratio.

**FIGURE 1:**
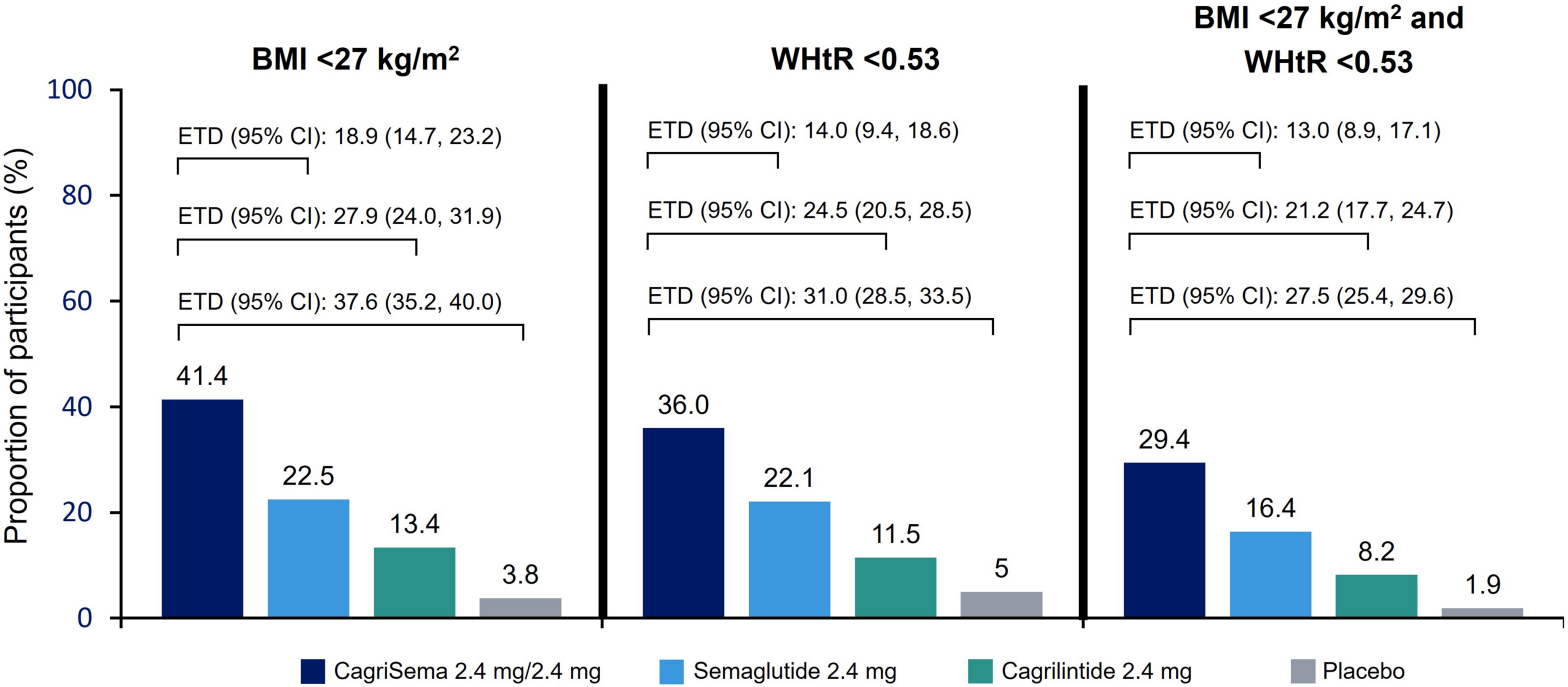
The proportion of participants achieving BMI < 27 kg/m^2^ and/or WHtR < 0.53 targets at week 68 in REDEFINE 1. Data are from the trial product estimand. Bars show estimates from statistical analysis. ETDs and corresponding CIs are from the statistical analysis. BMI: body mass index; CI: confidence interval; ETD: estimated treatment difference; WHtR: waist‐to‐height ratio.

**FIGURE 2:**
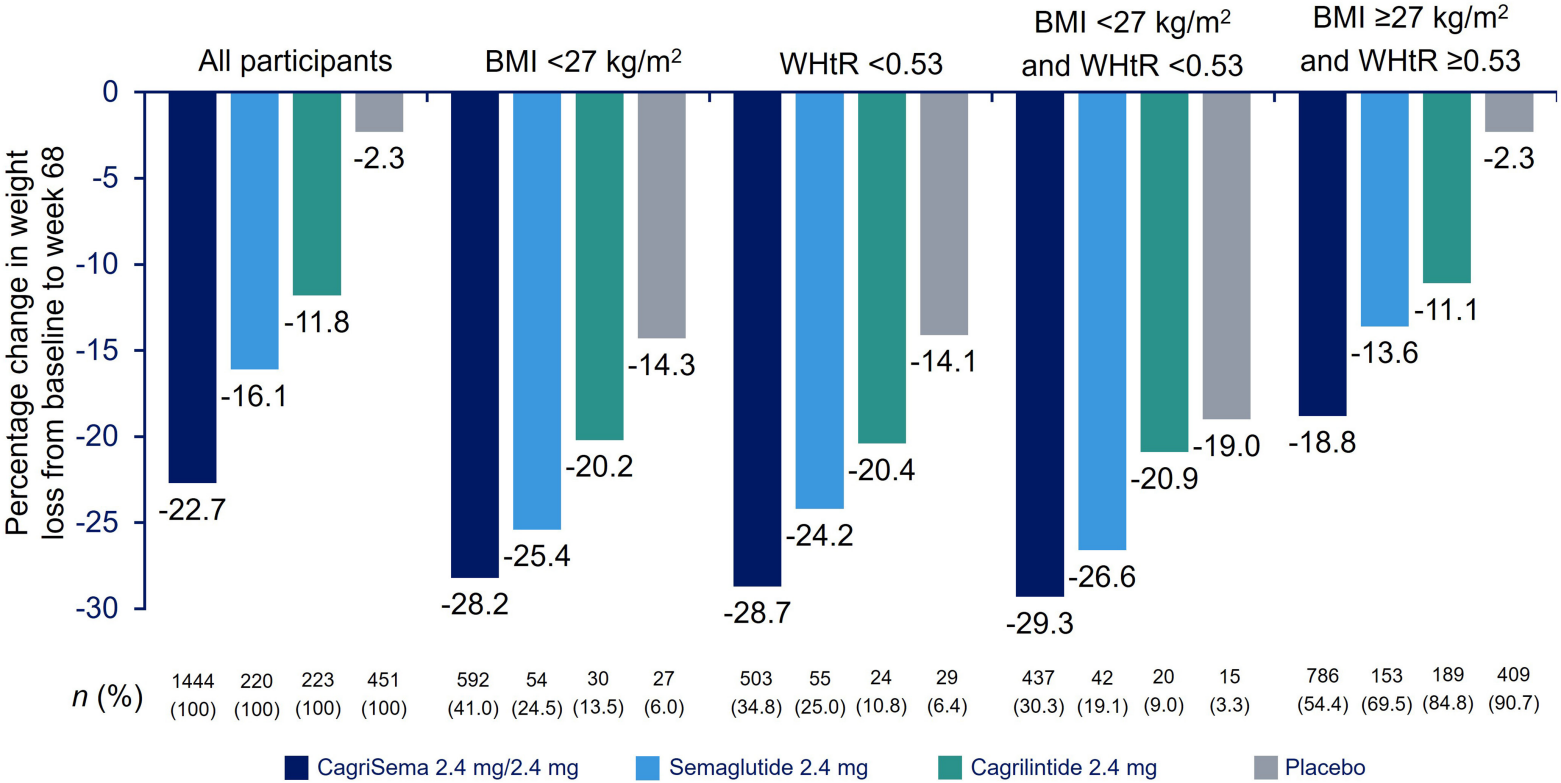
Percentage weight loss at week 68 by achievement of anthropometric targets. Data for “All participants” are from the trial product estimand. Data are observed data from the on‐treatment period showing treatment completers only. Analysis was based on a marginal logistic regression model including data on treatment without rescue medication. BMI: body mass index; WHtR: waist‐to‐height ratio.

On the basis of the trial product estimand, the ETD (95% CI) in the proportion of participants receiving CagriSema 2.4 mg/2.4 mg or placebo achieving a BMI < 27 kg/m^2^ at week 68 was 37.6 (35.2, 40.0). The corresponding ETD (95% CI) for participants achieving a WHtR < 0.53 was 31.0 (28.5, 33.5), and for participants achieving both BMI < 27 kg/m^2^ and WHtR < 0.53: 27.5 (25.4, 29.6) (Figure 1). Overall, the mean percent weight change from baseline to week 68 was −22.7% with CagriSema 2.4 mg/2.4 mg, −16.1% with semaglutide 2.4 mg, −11.8% with cagrilintide 2.4 mg and −2.3% with placebo [20]; the percentage weight loss at week 68 was greater in participants who achieved BMI < 27 kg/m^2^ and/or WHtR < 0.53 targets compared with all participants and those who did not reach treatment targets (Figure 2). Reductions in percentage weight loss at week 68 were consistently greater in CagriSema 2.4 mg/2.4 mg Versus semaglutide 2.4 mg, cagrilintide 2.4 mg and placebo groups, regardless of whether anthropometric targets were reached; in participants who received CagriSema 2.4 mg/2.4 mg, percentage weight loss at week 68 was 29.3% in individuals who achieved both BMI and WHtR targets Versus 18.8% in those who did not achieve both targets (Figure 2).

The proportion of individuals reaching BMI and/or WHtR anthropometric targets was lower in people with a baseline BMI > 35 kg/m^2^ compared with those with a BMI ≤ 35 kg/m^2^, across all treatment groups (Figure S2). In participants who received CagriSema 2.4 mg/2.4 mg, the proportion reaching both anthropometric targets was 59.4% in those with a starting BMI ≤ 35 kg/m^2^ versus 11.4% in those with a BMI > 35 kg/m^2^.

Similarly, the reduction in waist circumference from baseline to week 68 was greater in participants who achieved BMI < 27 kg/m^2^ and/or WHtR < 0.53 targets Versus those who did not. Reductions in waist circumference were greatest in participants who received CagriSema 2.4 mg/2.4 mg Versus monotherapies. In participants who received CagriSema 2.4 mg/2.4 mg, the reduction in waist circumference was −24.5 cm in individuals who achieved both anthropometric targets Versus 16.6 cm in those who did not (Figure S3).

### 3.3 Cardiometabolic Outcomes

**FIGURE 3:**
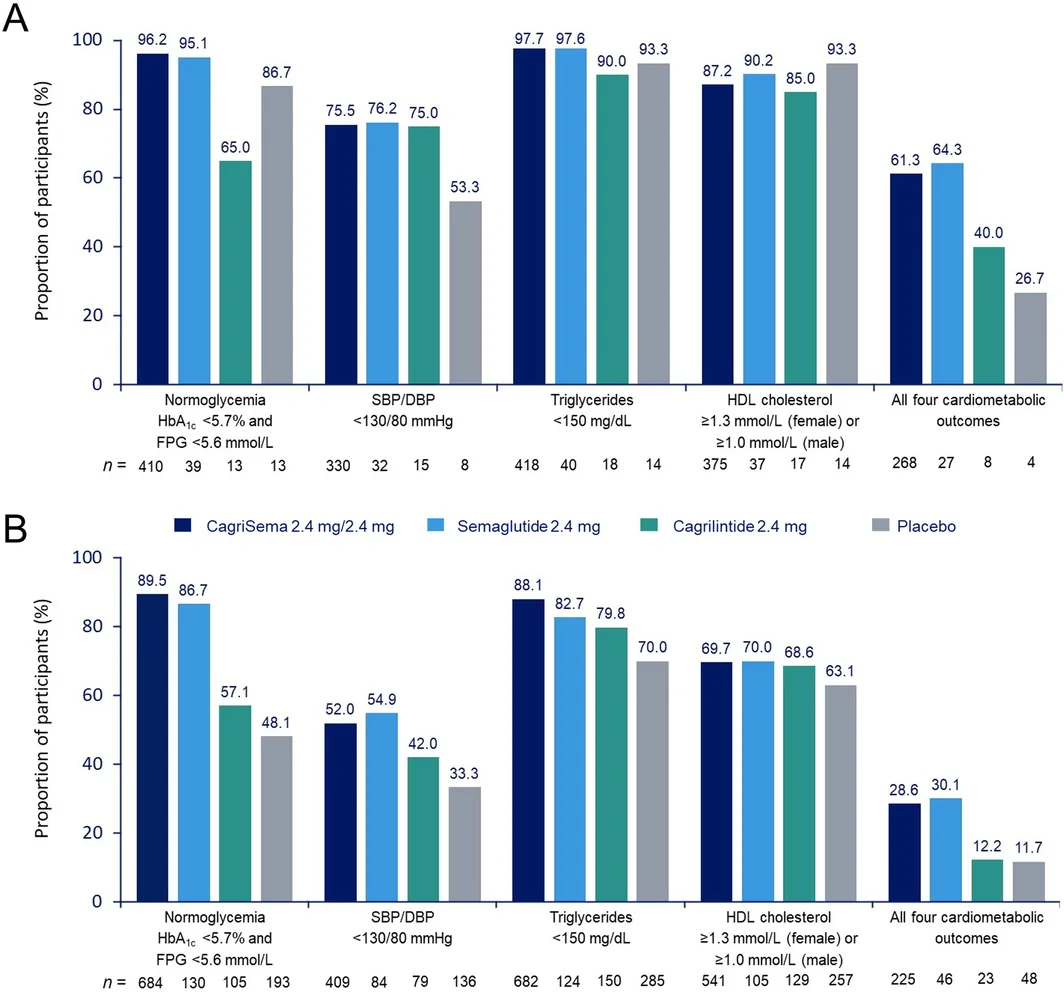
Maintenance or normalisation of cardiometabolic outcomes in participants who achieved both BMI < 27 kg/m^2^ and WHtR < 0.53 targets (A), and participants who did not achieve BMI < 27 kg/m^2^ and WHtR < 0.53 targets (B). Data are observed data from the on‐treatment period showing treatment completers only. BMI, body mass index; DBP, diastolic blood pressure; FPG, fasting plasma glucose; HbA_1c_, glycated haemoglobin; HDL, high‐density lipoprotein; SBP, systolic blood pressure; WHtR, waist‐to‐height ratio.

CagriSema 2.4 mg/2.4 mg led to a numerically higher proportion of participants achieving individual, and all, cardiometabolic outcomes compared with cagrilintide 2.4 mg and placebo, in participants who both did and did not achieve anthropometric targets (Figure 3). Across all treatment groups, a greater proportion of individuals who achieved BMI and/or WHtR targets reached cardiometabolic outcomes compared with those who did not attain those targets (Figure 3). In participants who received CagriSema 2.4 mg/2.4 mg, achievement of normoglycaemia was observed in 96.2% of participants who achieved both BMI and WHtR targets, Versus 89.5% in participants who achieved neither target. For other cardiometabolic outcomes, corresponding comparisons in participants receiving CagriSema 2.4 mg/2.4 mg who did or did not achieve both targets were 75.5% Versus 52.0% for SBP/DBP 130/80 mmHg, 97.7% Versus 88.1% for triglycerides < 150 mg/dL, and 87.2% Versus 69.7% for HDL cholesterol ≥ 1.0 mmol/L (male) or ≥ 1.3 mmol/L (female) (Figure 3). Across all treatment groups, the proportion of participants who achieved all four cardiometabolic outcomes was notably higher in those who achieved both anthropometric targets compared with those who did not; in participants who received CagriSema 2.4 mg/2.4 mg, this corresponded to 61.3% Versus 28.6%, respectively (Figure 3). Similarly, treatment with CagriSema 2.4 mg/2.4 mg led to a greater proportion of participants achieving the hsCRP outcome of < 2 mg/L, and of all four cardiometabolic outcomes plus the hsCRP outcomes, compared with cagrilintide 2.4 mg and placebo, in participants who achieved and did not achieve BMI and WHtR targets (Figure S4).

### 3.4 Anthropometric Treatment Targets and Change in Body Weight (%) for Maintenance or Achievement of All Four Cardiometabolic Outcomes

**FIGURE 4:**
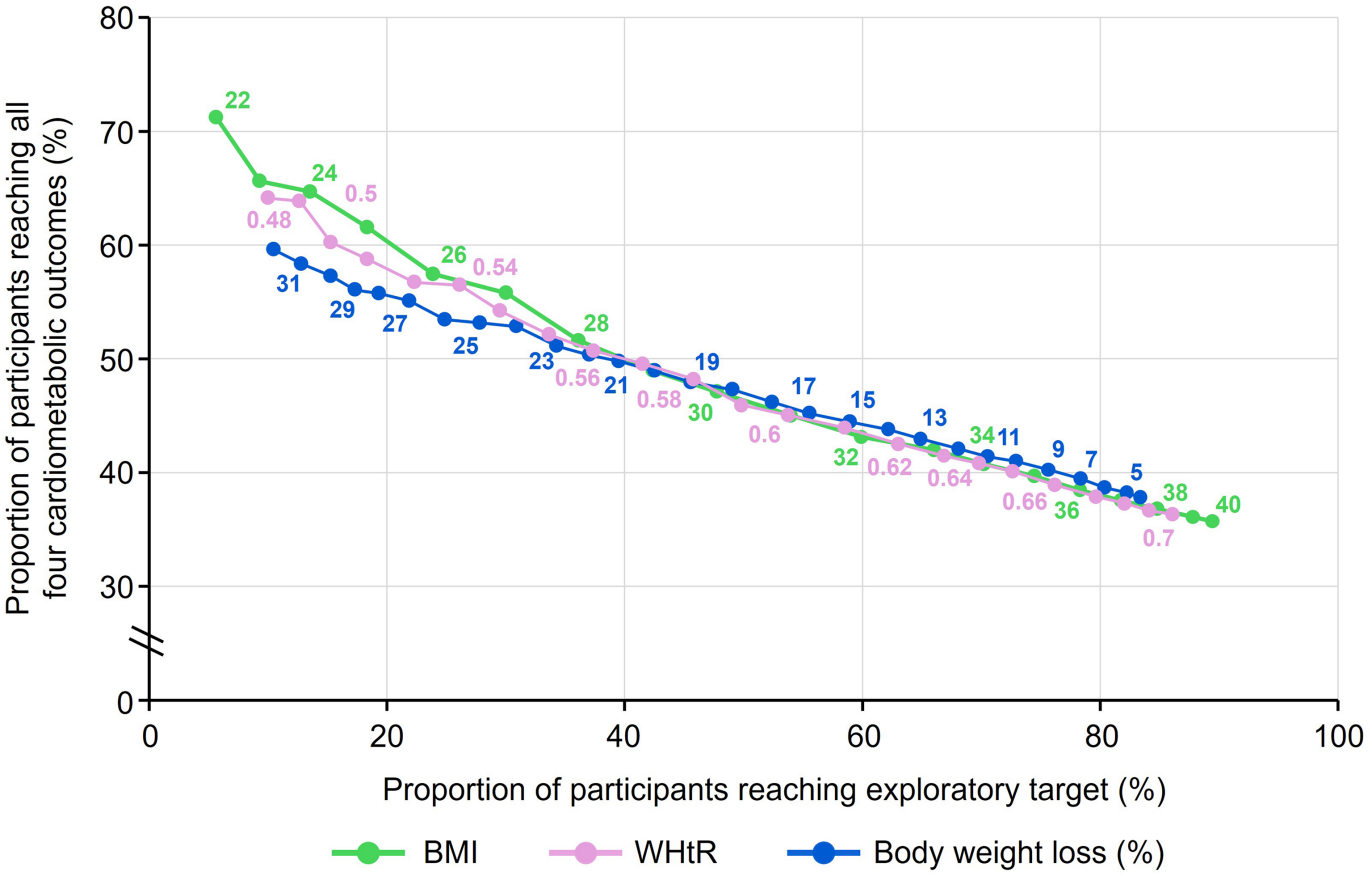
Comparison of treatment target performance—pooled analysis. Data are observed data from the on‐treatment period showing treatment completers only. The *x* axis corresponds to the proportion of participants who achieved different cutoffs for BMI, WHtR or percentage body weight targets, with the numbers on the Figure indicating reference values for each of these three groups. The y axis presents the proportion of participants who achieved all four cardiometabolic targets (HbA_1c_ < 5.7%) and FPG < 5.6 mmol/L (100 mg/dL); SBP < 130 mmHg and DBP < 80 mmHg; triglycerides < 1.7 mmol/L (< 150 mg/dL); HDL cholesterol ≥ 1.0 mmol/L (male) or ≥ 1.3 mmol/L (female). BMI, body mass index; DBP, diastolic blood pressure; FPG, fasting plasma glucose; HDL, high‐density lipoprotein; SBP, systolic blood pressure; WHtR, waist‐to‐height ratio.

In a pooled analysis of all participants, both BMI and WHtR performed similarly as a marker for reaching all four cardiometabolic outcomes (Figure 4). While percentage weight loss performed comparably with BMI and WHtR targets at lower cut‐off values, there was a separation in efficacy for reaching all four cardiometabolic outcomes at higher cut‐offs: in the 22% of participants who reached both BMI < 27 kg/m^2^ and WHtR < 0.53 targets, 60% met all four cardiometabolic outcomes; in the 28% of participants who achieved a ≥ 25% body weight loss, 53% met all four targets.

## 4 Discussion

In this secondary *post hoc* analysis of the REDEFINE 1 trial of people with overweight or obesity, a greater proportion of participants in the CagriSema 2.4 mg/2.4 mg group reached BMI < 27 kg/m^2^ and WHtR < 0.53 targets compared with those in the semaglutide 2.4 mg, cagrilintide 2.4 mg or placebo groups. Participants who reached BMI and/or WHtR targets had an increased rate of maintaining or achieving cardiometabolic outcome thresholds (glycaemia, triglycerides, HDL cholesterol and blood pressure); however, given the *post hoc* design, these findings should be interpreted as associations requiring prospective confirmation. Furthermore, reaching both BMI and WHtR targets presented a modest improvement in cardiometabolic outcomes compared with participants reaching ≤ 25% weight loss targets. These data highlight the link between new anthropometric targets in the clinical management of obesity, attained by nearly a third of participants who received CagriSema 2.4 mg/2.4 mg, and the near normalisation of cardiometabolic risk factors.

A treat‐to‐target approach is routinely employed for chronic diseases, such as T2D, hypertension, and dyslipidaemia, with treatment targets informing clinical decisions regarding the amelioration or reduced risk of adverse outcomes [1, 3, 4]. Moreover, treat‐to‐target also transitions clinical trials from simple, static interventions to a more dynamic approach with individualised treatment targets that are responsive to evolving health status [21, 22]. In obesity management, the clinical goal of reaching a disease state with a low risk of developing cardiometabolic ORCDs may be more effectively met by defining absolute anthropometric treatment targets known to be associated with cardiometabolic risk and ORCDs [19]. Following analysis of real‐world data from a UK primary care database, Busetto et al. identified that reaching absolute BMI ≤ 27 kg/m^2^ and WHtR ≤ 0.53 was a better marker of low risk of ORCDs than relative changes such as percentage weight loss, and proposed these targets as an initial step towards more individualised and effective obesity management [19]. In the present analysis, anthropometric treatment targets of < 27 kg/m^2^ and WHtR < 0.53 were employed in line with using “<”, rather than “≤”, for defining goals in individuals with T2D [23], or with hypertension [24]. Furthermore, the same BMI and WHtR targets were used in a recent analysis of tirzepatide, and semaglutide 2.4 mg in the SURMOUNT‐5 trial [25].

Here, the proportions of participants reaching BMI, WHtR, or both targets were consistently greater in the CagriSema 2.4 mg/2.4 group Versus semaglutide 2.4 mg. However, the maintenance or normalisation of cardiometabolic factors in individuals who achieved anthropometric targets was similar across both CagriSema 2.4 mg/2.4 and the semaglutide 2.4 mg treatment groups, while this was less uniform with regards to percentage weight loss. Maintenance or normalisation of cardiometabolic outcomes in individuals reaching anthropometric targets who received cagrilintide 2.4 mg was comparable to the other treatment arms, except for a notable reduction in the proportion achieving normoglycaemia, possibly due to the proven incretin effects observed with GLP‐1 receptor agonists, such as semaglutide [26]. The BMI—and WHtR‐based treatment targets implemented here appeared particularly effective at distinguishing participants who maintained or normalised all four cardiometabolic outcomes from those who did not, with both BMI < 27 kg/m^2^ and WHtR < 0.53 treatment targets performing similarly.

While percentage body weight reduction remains a fair indicator of mean response in a population with obesity, our results indicate that reaching the absolute anthropometric treatment targets may provide additional value for determining a state of low disease activity, particularly for participants with a BMI: 27–34.9 kg/m^2^ [27]. Furthermore, our data indicate that reaching anthropometric targets is a function of treatment response and not merely the result of having started closer to the goal from the beginning, thereby further validating the targets as treatment goals, even when using percentage weight loss. In addition, the near 30% weight loss observed in participants who received CagriSema 2.4 mg/2.4 mg and achieved both anthropometric targets suggests that conducting a study with a treat‐to‐target design may result in greater percentage weight loss. The introduction of anthropometric treatment targets complements other relevant targets unrelated to weight loss or anthropometric data, such as physical and mental‐related quality of life or the patient's overall satisfaction. However, fixed, absolute anthropometric targets may be less attainable for individuals with high baseline BMI (> 35 kg/m^2^) and/or WHtR. In such individuals, combination therapy with modalities such as metabolic bariatric surgery may be required [28]. Percentage weight loss, waist circumference reduction, cardiometabolic improvement, functional outcomes, and patient‐reported outcomes also remain a relevant measure of success to be pursued in future studies. Indeed, some individuals in this study achieved maintenance or normalisation of cardiometabolic parameters without reaching anthropometric targets. Both BMI and WHtR should be considered when defining treatment targets for ORCDs risk reduction given the substantial associated health and economic burden [29, 30, 31].

The descriptive nature of these secondary analyses of REDEFINE 1 supports the need for adequately powered studies to investigate whether reaching these targets is associated with meeting cardiometabolic outcomes using more formal predictive performance metrics to statistically compare the performance of anthropometric and percentage weight loss targets. Such studies should include assessing the impact of baseline demographic factors such as BMI and waist circumference that may affect the proportion of participants reaching targets. Our data indicate that treatment targets lower than 27 kg/m^2^ for BMI and 0.53 for WHtR would lead to a higher proportion of participants reaching all four cardiovascular outcomes. As such, future research should also investigate a treat‐to‐target approach as a clinical strategy, not solely as endpoints in a fixed‐dosing regimen, with the ultimate aim of improving shared decision‐making between the physician and patient. The present findings should also be interpreted in the context of lifestyle support accompanying pharmacotherapy. Although highly effective obesity medications can produce substantial weight reduction, structured lifestyle counselling, individualised diet and exercise planning, and regular follow‐up remain important components of obesity care and need to be considered in future treat‐to‐target obesity trials.

Strengths of this study included the large sample size, the comprehensive collection of a wide range of study data over a 68‐week period allowing clinically meaningful evaluation and the ability to compare across CagriSema 2.4 mg/2.4 mg, semaglutide 2.4 mg and cagrilintide 2.4 mg treatment groups. Limitations include the *post hoc* nature of the study, the reduced capacity for statistical comparisons and the fact that patients were not treated‐to‐target. Furthermore, the anthropometric targets used in this study are derived from an adult population aged 18 to 60 years [19], and therefore may not be applicable to older age groups. Additional limitations include a predominantly female and White population.

Absolute treatment targets are needed to guide personalised obesity care and better predict individual clinical benefits. In this *post hoc* analysis of the REDEFINE 1 study, a greater proportion of participants in the CagriSema 2.4 mg/2.4 mg group reached the BMI and WHtR targets compared with semaglutide 2.4 mg, cagrilintide 2.4 mg, and placebo. In the subset of participants reaching these anthropometric treatment targets, > 60% also met cardiometabolic outcomes, consistent with a low‐risk disease state. Given the *post hoc* nature of this analysis, these findings should be interpreted as hypothesis‐generating and supportive of further prospective validation of BMI—and WHtR‐based treatment targets, rather than as definitive evidence for their immediate use as treatment escalation or de‐escalation thresholds.

## Author Contributions

All authors had full access to all data in the study and had final responsibility for the decision to submit for publication. All authors contributed equally to the data interpretation and manuscript writing, approved the final version of the manuscript, and vouch for data accuracy and fidelity to the protocol.

## Funding

Novo Nordisk A/S Was the Study Sponsor, and Was Responsible for the Study Design, Preparing the Protocol, and Performing the Statistical Analyses. This Article Was Drafted Under the Guidance of the Authors Who Were Responsible for All Decisions Regarding Publication, With Medical Writing and Editorial Support Paid for by the Funder.

## Conflicts of Interest

L.B. has received speaker fees from PronoKal, Rhythm Pharmaceuticals; has received consulting fees from Amgen, Boehringer Ingelheim, Bruno Farmaceutici, Eli‐Lilly, Novo Nordisk, Pfizer, Recordati, Regeneron, and Roche. W.T.G. has served as consultant on advisory boards for Boehringer Ingelheim, Eli Lilly, Novo Nordisk, Pfizer, Fractyl Health, Zealand, Genentech/Roche, TERNS Pharmaceuticals, Neurocrine, Keros Therapeutics, Gan & Lee, Corcept, Metsera, Graviton Bioscience, AbbVie, Madrigal, i2o Therapeutics, and Regeneron, and as site principal investigator for multi‐centered clinical trials sponsored by his university and funded by Novo Nordisk, Eli Lilly, Zealand, Genentech/Roche, TERNS Pharmaceuticals, Kailera, and Viking Therapeutics. D.B.H. has acted as a consultant for Amgen, AstraZeneca, Boehringer Ingelheim, Eli Lilly, Novo Nordisk and Zealand Pharma, and as a speaker for Eli Lilly and Novo Nordisk; and has received institutional research funding from Eli Lilly, KVK Tech, Novo Nordisk and WeightWatchers. B.M. reports speakers fees from Novo Nordisk, Eli Lilly, Amgen, and AstraZeneca, consultancy fees from AstraZeneca, Eli Lilly, Novo Nordisk, and Boehringer Ingelheim, and is a shareholder in Reset Health. C.l.R. reports grants from the EU Innovative Medicine Initiative, Irish Research Council, Science Foundation Ireland, AnaBio, and the Health Research Board. He serves on advisory boards and speakers panels of Novo Nordisk, Roche, Herbalife, Morphic Medical, Eli Lilly, Johnson & Johnson, Gila, Irish Life Health, Boehringer Ingelheim, Currax, Zealand Pharma, Keyron, AstraZeneca, Arrowhead Pharma, Amgen, AbbVie, Metsera, Nymble, Olympus, and Rhythm Pharma. C.l.R. is the Chair of the Irish Society for Nutrition and Metabolism. C.l.R. received stock options as payment for scientific advisory board functions from Metsera and Nymble. C.l.R. provides obesity clinical care in the My Best Weight clinic and Beyond BMI clinic and is a co‐owner of these clinics. V.S. is an employee of Novo Nordisk A/S. C.O.C., M.H.C., and C.P.M. are shareholders and employees of Novo Nordisk A/S.

## Supporting information


**Table S1:** Demographics and baseline characteristics by anthropometric target group.
**Figure S1:** Study design.
**Figure S2:** The proportion of participants achieving BMI < 27 kg/m^2^ and/or WHtR < 0.53 targets at week 68 in REDEFINE 1 in individuals with a BMI ≤ 35 kg/m2 at baseline (A), and a BMI > 35 kg/m^2^ (B).
**Figure S3:** Change in waist circumference at week 68 by achievement of anthropometric targets.
**Figure S4:** Normalisation of hsCRP and all cardiometabolic outcomes plus hsCRP in participants who did, and did not, achieve BMI < 27 kg/m^2^ and WHtR < 0.53 targets.

## Data Availability

An authorised researcher can request access to clinical study data by submitting a research proposal for review and approval by Novo Nordisk and an internal independent review panel. Requests are usually considered after the research is finished and the main results have been published. If the research supports a regulatory application, requests will be considered after the product and its intended use are approved in both the EU and the USA. Participants' clinical data will be anonymised, following an approved internal process, before data are shared with external third parties. For details on how to request access to clinical data, visit novonordisk‐trials.com.
