# Supplementary material for: Efficacy of CagriSema for Reaching Anthropometric Treatment Targets and Cardiometabolic Outcomes: A Secondary, Post hoc Analysis of REDEFINE 1
Source: Diabetes Obes Metab. 2026 Jul 26;28(10):9317–25. doi: 10.1111/dom.71134 (PMC13538786; doi:10.1111/dom.71134)
Supplement: Supplementary file 1 [file DOM-28-9317-s001.docx]

**Efficacy of CagriSema for reaching anthropometric treatment targets and cardiometabolic outcomes: a secondary, *post hoc* analysis of REDEFINE 1**

**Running header:** Anthropometric treatment targets and cardiometabolic outcomes in REDEFINE 1

Luca Busetto,^1^ Cynthia Osorto Contreras,^2^ Mikkel Hovden Christensen,^2^ W. Timothy Garvey,^3^ Deborah B. Horn,^4^ Barbara McGowan,^5^ Claire Prener Miller,^2^ Volker Schnecke,^2^ Carel W. le Roux^6,7^

^1^Department of Medicine, University of Padova, Padova, Italy; ^2^Novo Nordisk A/S, Søborg, Denmark; ^3^University of Alabama at Birmingham, Birmingham, AL, USA; ^4^Center for Obesity Medicine and Metabolic Performance, University of Texas McGovern Medical School, Houston, TX, USA; ^5^Guy’s and St. Thomas’ NHS Foundation Trust, London, UK; ^6^Diabetes Complications Research Centre, Conway Institute, University College Dublin, Dublin, Ireland; ^7^Diabetes Research Centre, Ulster University, Coleraine, UK **Contents**

Table S1 Demographics and baseline characteristics by anthropometric target group………….3

Figure S1 Study design………………………………………………………………………………….5

Figure S2 The proportion of participants achieving BMI <27 kg/m^2^ and/or WHtR <0.53 targets at week 68 in REDEFINE 1 in individuals with a BMI ≤ 35 kg/m2 at baseline (A), and a BMI >35 kg/m^2^ (B)………………………………………………………………………………….………………..6

Figure S3 Change in waist circumference at week 68 by achievement of anthropometric targets……………………………………………………………………………………………………...7

Figure S4 Normalisation of hsCRP and all cardiometabolic outcomes plus hsCRP in participants who did, and did not, achieve BMI <27 kg/m^2^ and WHtR <0.53 targets………………..…………..8

**SUPPLEMENTARY TABLE S1** Demographics and baseline characteristics by anthropometric target group

|  | **CagriSema 2.4 mg/2.4 mg** | | **Semaglutide 2.4 mg** | | **Cagrilintide 2.4 mg** | | **Placebo** | |
| --- | --- | --- | --- | --- | --- | --- | --- | --- |
|  | BMI <27 kg/m^2^ and WHtR <0.53 | BMI ≥27 kg/m^2^ and WHtR ≥0.53 | BMI <27 kg/m^2^ and WHtR <0.53 | BMI ≥27 kg/m^2^ and WHtR ≥0.53 | BMI <27 kg/m^2^ and WHtR <0.53 | BMI ≥27 kg/m^2^ and WHtR ≥0.53 | BMI <27 kg/m^2^ and WHtR <0.53 | BMI ≥27 kg/m^2^ and WHtR ≥0.53 |
|  | *n* (%) | *n* (%) | *n* (%) | *n* (%) | *n* (%) | *n* (%) | *n* (%) | *n* (%) |
| Number of participants | 437 | 786 | 42 | 153 | 20 | 189 | 15 | 409 |
| Age (years) |  |  |  |  |  |  |  |  |
| 18–64 | 416 (95.2) | 739 (94.0) | 40 (95.2) | 144 (94.1) | 17 (85.0) | 180 (95.2) | 15 (100) | 383 (93.6) |
| 65–75 | 20 (4.6) | 43 (5.5) | 2 (4.8) | 7 (4.6) | 3 (15.0) | 9 (4.8) | 0 | 23 (5.6) |
| ≥75 | 1 (0.2) | 4 (0.5) | 0 | 2 (1.3) | 0 | 0 | 0 | 3 (0.7) |
| Sex |  |  |  |  |  |  |  |  |
| Female | 341 (78.0) | 466 (59.3) | 34 (81.0) | 86 (56.2) | 15 (75.0) | 120 (63.5) | 9 (60.0) | 260 (63.6) |
| Male | 96 (22.0) | 320 (40.7) | 8 (19.0) | 67 (43.8) | 5 (25.0) | 69 (36.5) | 6 (40.0) | 149 (36.4) |
| Ethnic origin |  |  |  |  |  |  |  |  |
| Hispanic or Latino | 26 (5.9) | 102 (13.0) | 3 (7.1) | 18 (11.8) | 1 (5.0) | 15 (7.9) | 1 (6.7) | 37 (9.0) |
| Not Hispanic or Latino | 384 (87.9) | 664 (84.5) | 38 (90.5) | 132 (86.3) | 19 (95.0) | 168 (88.9) | 13 (86.7) | 359 (87.8) |
| Not reported | 27 (6.2) | 20 (2.5) | 1 (2.4) | 3 (2.0) | 0 | 6 (3.2) | 1 (6.7) | 13 (3.2) |
| Race |  |  |  |  |  |  |  |  |
| American Indian or  Alaska Native | 0 | 3 (0.4) | 0 | 0 | 0 | 1 (0.5) | 0 | 1 (0.2) |
| Asian | 113 (25.9) | 119 (15.1) | 10 (23.8) | 19 (12.4) | 6 (30.0) | 32 (16.9) | 4 (26.7) | 104 (25.4) |
| Black or African American | 7 (1.6) | 44 (5.6) | 1 (2.4) | 5 (3.3) | 0 | 10 (5.3) | 1 (6.7) | 19 (4.6) |
| Native Hawaiian or other Pacific Islander | 0 | 2 (0.3) | 0 | 0 | 0 | 0 | 0 | 0 |
| White | 292 (66.8) | 597 (76.0) | 31 (73.8) | 125 (81.7) | 14 (70.0) | 142 (75.1) | 10 (66.7) | 272 (66.5) |
| Multiple | 3 (0.7) | 5 (0.6) | 0 | 1 (0.7) | 0 | 0 | 0 | 0 |
| Not reported | 20 (4.6) | 14 (1.8) | 0 | 3 (2.0) | 0 | 4 (2.1) | 0 | 13 (3.2) |
| Missing | 2 (0.5) | 2 (0.3) | 0 | 0 | 0 | 0 | 0 | 0 |
| BMI, kg/m^2^ |  |  |  |  |  |  |  |  |
| <30 | 67 (15.3) | 6 (0.8) | 11 (26.2) | 3 (2.0) | 5 (25.0) | 4 (2.1) | 7 (46.7) | 24 (5.9) |
| 30–<35 | 258 (59.0) | 99 (12.6) | 24 (57.1) | 37 (24.2) | 12 (60.0) | 54 (28.6) | 6 (40.0) | 129 (31.5) |
| 35–<40 | 91 (20.8) | 243 (30.9) | 7 (16.7) | 57 (37.3) | 3 (15.0) | 67 (35.4) | 1 (6.7) | 133 (32.5) |
| ≥40 | 21 (4.8) | 438 (55.7) | 0 | 56 (36.6) | 0 | 64 (33.9) | 1 (6.7) | 123 (30.1) |

BMI, body mass index; WHtR, waist-to-height ratio.

**SUPPLEMENTARY FIGURE S1** Study design.

^†^Treatment received as an adjunct to lifestyle intervention (-500 kcal/day diet plus 150 min/week physical activity).

Garvey WT, et al. N Engl J Med. 2025;393(7):635–647.

**SUPPLEMENTARY FIGURE S2** The proportion of participants achieving BMI <27 kg/m^2^ and/or WHtR <0.53 targets at week 68 in REDEFINE 1 in individuals with a BMI ≤ 35 kg/m^2^ at baseline (A), and a BMI >35 kg/m^2^ (B).

Data are from the trial product estimand. Bars show estimates from statistical analysis. ETD’s and corresponding CI’s are from the statistical analysis.

BMI, body mass index; CI, confidence interval; ETD, estimated treatment difference; WHtR, waist-to-height ratio.

**SUPPLEMENTARY FIGURE S3** Change in waist circumference at week 68 by achievement of anthropometric targets.

Data for “All participants” are from the trial product estimand for treatment completers only. Analysis was based on marginal logistic regression model including data on treatment without rescue medication.

BMI, body mass index; WHtR, waist-to-height ratio.

**SUPPLEMENTARY FIGURE S4** Maintenance or normalisation of hsCRP and all cardiometabolic outcomes plus hsCRP in participants who did, and did not, achieve BMI <27 kg/m^2^ and WHtR <0.53 targets.

Data are observed data from the on-treatment period showing treatment completers only.

BMI, body mass index; hsCRP, high-sensitivity C-reactive protein; WHtR, waist-to-height ratio.
